# Stakeholders’ experiences of the public health research process: time to change the system?

**DOI:** 10.1186/s12961-020-00599-5

**Published:** 2020-07-18

**Authors:** Yvonne Laird, Jillian Manner, Louise Baldwin, Ruth Hunter, John McAteer, Sarah Rodgers, Chloë Williamson, Ruth Jepson

**Affiliations:** 1 Sydney School of Public Health, Prevention Research Collaboration, Charles Perkins Centre, University of Sydney, Sydney, Australia; 2 Scottish Collaboration for Public Health Research and Policy, School of Health in Social Science, University of Edinburgh, Edinburgh, United Kingdom; 3 School of Public Health and Social Work, Queensland University of Technology (QUT), Institute of Health and Biomedical Innovation, Brisbane, Australia; 4 Centre for Public Health, Queen’s University Belfast, Belfast, United Kingdom; 5 Department of Public Health and Policy, University of Liverpool, Liverpool, United Kingdom; 6 Physical Activity for Health Research Centre, Institute of Sport, Physical Education and Health Sciences, Moray House School of Education and Sport, University of Edinburgh, Edinburgh, United Kingdom

**Keywords:** co-production, collaboration, interdisciplinary, action research, partnerships

## Abstract

**Background:**

The importance of engaging stakeholders in the research process is well recognised. Whilst engagement is important, guidelines and practices vary for how stakeholders should be involved in research and how to facilitate effective collaborative relationships.

**Methods:**

This study aimed to explore the perspectives and experiences of stakeholders involved in the policy and practice area of outdoor space and non-communicable disease prevention. Stakeholders interviewed included academics, practitioners, policy-makers, knowledge brokers and a funder.

**Results:**

The findings suggest that stakeholders had positive experiences when engaged meaningfully in the research process, where research projects were carefully planned and managed with attention to context and culture, and where the research team was effective, respectful and communicative. These factors help to facilitate the translation of research into policy and practice. However, multiple challenges of collaborative research were identified which related to structural and systemic challenges, building and maintaining relationships, use and collection of data and information, cultural perceptions of research and research generation, and getting evidence into action. Participants felt that changing the funding system, exploring more collaborative research methodologies, improved research translation, and more effective collaborative relationships at all stages of the research process could address some of these challenges.

**Conclusions:**

The findings highlight that, whilst stakeholder engagement in research was considered important, structural, cultural and individual practices impacted how this worked in practice. Identifying and testing solutions to address these challenges could improve synergies between research, policy, and practice and lead to the production of impactful research that reduces wastage of public funding, improves implementation of findings and ultimately improves public health outcomes.

## Background

Non-communicable diseases (NCDs) are a major public health concern, responsible for almost 70% of all deaths worldwide [1]. Tobacco consumption, physical inactivity, alcohol misuse and unhealthy diet are risk factors for the rise in NCDs [2]. Despite efforts from policy-makers, practitioners and researchers to tackle NCDs, viable, sustainable, population-wide solutions have not yet been identified. Coupled with this, an estimated 85% of medical research expenditure is wasted through failure to disseminate, not publishing findings in an accessible format, not building on previous research, and failure to align research to policy, practitioner and public need [3, 4]. If meaningful solutions to tackle NCDs are to be found, there is a need to identify new ways of working across research, policy and practice that reduces research wastage.

One such mechanism of addressing this lack of progress in NCD prevention is arguably through improving synergies between research, policy and practice systems. These synergies can potentially be enhanced through stakeholder engagement, which refers to the myriad of ways that stakeholders can be involved in the design, conduct and dissemination of research. Recent years have seen considerable growth in academic interest in collaborative engagement with policy and practice, in part due to the emphasis placed on impact by research quality assessments [5, 6]. Major funding bodies recommend the involvement of non-academics, which includes stakeholders not affiliated with academic institutions (e.g. members of the public, policy-makers, practitioners), in research projects and grant applications. Some funding bodies now offer dedicated funding streams for public engagement (e.g. the Wellcome Trust in the United Kingdom), increasing scholarly recognition of the potential for participatory research methods such as citizen science and community-based participatory research [7, 8]. Additionally, a number of national and international networks dedicated to stakeholder engagement in research have been established (e.g. https://www.publicengagement.ac.uk, https://ecsa.citizen-science.net/, and http://coproductionscotland.org.uk).

Despite the growth of collaborative research, meaningful involvement of stakeholders in public health research, for example, as co-researchers, is not commonplace, with most research still predominantly conducted in academic institutions by researchers. In public health research, this lack of meaningful involvement seems at odds with principles of community engagement and intersectoral collaboration seen in public health practice. We argue there is a need to develop and embed (1) approaches to establishing and maintaining effective collaborative relationships with professionals in the research process and (2) approaches to meaningfully engage citizens in the research process such as citizen science and participatory action research. In this paper, we consider ways of enhancing the involvement and experiences of professional stakeholders in public health research.

Stakeholder engagement is important for public health science to ensure that planned or funded research is relevant and addresses key public health concerns for policy-makers, practitioners and the public. This engagement could involve identifying areas of need in local settings, prioritising research and providing input into the acceptability of research methods and tools. Such input increases the success of interventions, for example, by providing contextual information that can either impede or facilitate implementation. Research is more likely to be embedded in policy and practice when it is planned and conducted in conjunction with stakeholders, leading to enhanced research impact [9–11].

Challenges to stakeholder engagement include working with project partners who are geographically dispersed, management of divergent opinions across collaborators, power inequalities, competing priorities, less opportunities for face-to-face contact, partners who are less committed to stakeholder engagement, stakeholders who are not willing or interested in engaging in the research project, and striking a balance between the practical relevance of research and scientific merit [12–14]. System level barriers, such as conflicting priorities and timescales between researchers, policy-makers and practitioners, funders priorities, and the academic reward system have also been identified [15–17]. Facilitators to collaborative working include supportive organisational principles (e.g. setting clear objectives and plans for stakeholder engagement), fostering shared values to research and stakeholder engagement, building trust, provision of training, and practices that consider the roles and responsibilities of stakeholders at each stage of the research process (e.g. considering how stakeholder input can be analysed) [12, 18–21]. The quality of collaborative relationships, including the structure and process of collaborations, has also been identified as impacting partnership outcomes relating to good governance [14].

This research provides a starting point for developing effective collaborations with stakeholders and, whilst there is a growing body of research outlining the benefits of partnerships and collaborative methodological approaches (e.g. [13, 22–26]), there is an identified lack of guidance on forming and maintaining effective research partnerships [27], with existing evidence predominantly from the perspective of the academic. Understanding stakeholders’ experiences of engaging in the research process could identify strategies aimed at better facilitating positive engagement. This study aimed to explore the perspectives and experiences of a range of professional stakeholders involved in generating or using research in public health. This paper will specifically focus on outdoor space and NCD prevention to provide a focus for participants to discuss, but the findings are likely to be generalisable to other research areas in public health. The objectives are to understand (1) the challenges and enablers of stakeholder engagement in the research process and use of research evidence; (2) experiences of stakeholder engagement in the research process; and (3) the ways in which the research process could better facilitate stakeholder engagement and the use of research evidence.

## Methods

This qualitative interview study employed thematic analysis following the six-step approach outlined by Braun and Clarke [28] using a constructionist perspective. Thematic analysis enabled a rich theoretical insight into participants’ experiences of stakeholder engagement. The study was conceived by a team of academics and non-academic partners, based on prior experiences of co-production and collaborative working. The project participants (both academic and non-academic) shaped the direction of the research through providing informal feedback on the topic, the interview process and the initial findings. Ethical approval for this project was granted by the Usher Institute Research Ethics Committee at the University of Edinburgh (ethical approval code 1764). This study followed the procedures for reporting qualitative research outlined in the consolidated criteria for reporting qualitative research (COREQ) checklist [29].

### Sample and procedure

A purposive sample of 33 policy-makers, researchers, practitioners, funders, consultants and directors of public health involved in generating or using research in outdoor space and NCD prevention in the United Kingdom were invited to take part via an email invitation. Of these, 21 agreed and 20 participated in a semi-structured interview following receipt of informed written consent. This included six in-person interviews, conducted in a quiet area in the participants’ workplace, and 14 telephone interviews. Three researchers (YL, JM and CW), with training and experience in qualitative interviewing, conducted interviews between May and June 2018. Interviews were recorded using a digital audio recorder and lasted 22 to 54 minutes. The interviewers took detailed notes during interviews to aid reflection.

### Topic guide

A topic guide was prepared with questions designed to elicit open responses. Questions were structured around (1) participants’ experiences of using and generating research, (2) enablers of using or generating research, (3) challenges of using or generating research, (4) perspectives on what needs to change to facilitate stakeholder engagement, and (5) experiences of collaborative research. Questions were modified based on whether the participant was a researcher, policy-maker, funder or practitioner. The topic guide was pilot tested and, following this, minor adaptions were made to improve clarity.

### Data analysis

Interviews were transcribed verbatim, excluding identifiable information. Transcripts were read over carefully and checked for accuracy. The data were analysed inductively using the six-stage process of thematic analysis outlined by Braun and Clarke [28]; however, existing knowledge may also have influenced coding and theme-generation. Where possible, an iterative process was employed whereby interviews were conducted, transcribed and analysed, with the analysis informing subsequent data collection.

Coding was carried out by two researchers to facilitate reflection, discuss emerging findings and uncertainties, and to gain additional insights into the data. The first three transcripts were coded independently by two researchers. Following this, a coding framework was agreed between the researchers (Supplementary file 1). The coding framework was used as a guide for coding the remaining transcripts and was not prescriptive. Analysis was facilitated using NVivo 11 (QSR International). The coding of each transcript was cross-checked by another researcher (YL or JM), enabling additional reflexivity and the opportunity to discuss and explore uncertainties. The consistency of coding was checked between the initial and final transcripts. A total of 402 codes were initially identified and applied independently across the transcripts by two researchers. Codes that were similar were combined or grouped together to form overarching themes and sub-themes, with a final total of 60 codes. Any discrepancies were resolved through discussion with a third researcher. Data collection continued until the study authors were satisfied that the resulting data and analysis was sufficiently rich and adequately addressed the research questions. The final set of themes and sub-themes were discussed and agreed with the wider project team. A summary of the research findings was circulated to participants for comment. Any differences in opinion between academics and non-academics are outlined in the findings.

## Results

**Fig. 1:**
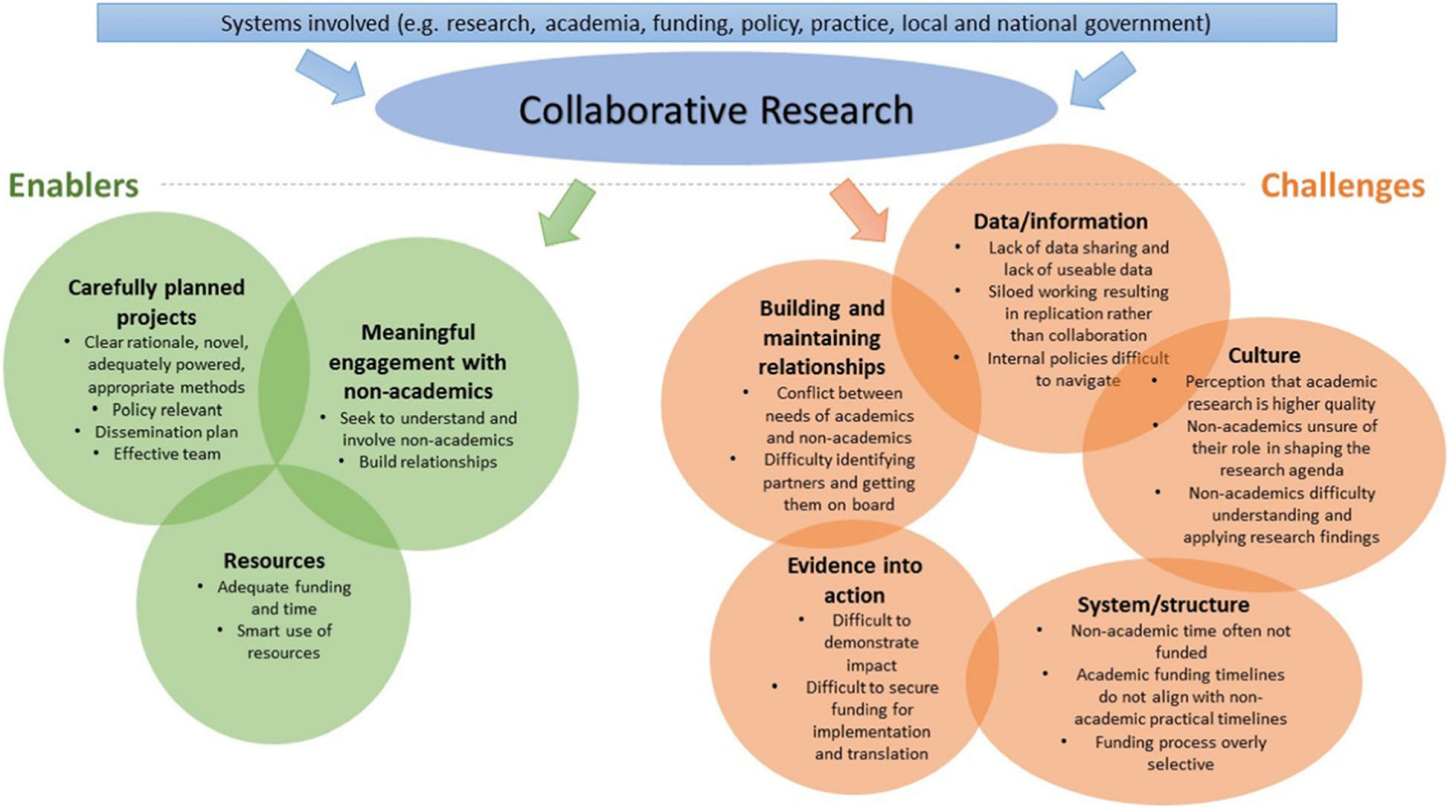
Challenges and enables of a collaborative research process

Participants included four senior academics, three policy-makers, nine practitioners, one funder, one consultant in public health, and two knowledge exchange experts (*n* = 20). Participants were based in universities, charities/non-profits, local and national government, the National Health Service (NHS) and government funded organisations in the United Kingdom. Some participants had multiple roles (e.g. responsibility for policy development and funding) and all participants (except the funder) had expertise in outdoor space and NCD prevention. Participants described factors that enabled engagement in the research process and use of research evidence, challenges and suggestions for change (Fig. 1). As demonstrated in Fig. 1, themes and sub-themes are not independent of each other and interact in a complex system.

### Enablers of stakeholder engagement in the research process

Enabling factors included carefully planned and sufficiently resourced research, with an effective team. Participants noted that these criteria were central to the generation of high-quality, collaborative research.

Meaningful engagement with non-academics was considered to play a vital role to ensure that research is policy relevant, sensitive to local contexts, and that findings are implemented into practice. Participants felt that this engagement should be co-operative and should seek to understand and involve non-academics. Participants also noted that collaborative research should be mutually beneficial, potentially facilitating new lines of thinking, providing access to participants and data, and strengthening funding applications for academics whilst helping non-academics to gain traction and profile, influence research agendas, and improve their practice and ways of working.

“*The more closely we can work with the research community the better able we are to be able to, on the one hand, to understand it ourselves. And on the other hand, to be able to support the research community in making sure that what they're producing is something that people outside of the research community are going to be able to understand. Whether that be the public or whether that be government departments.*” (Practitioner, participant 1)

There were mixed views as to how involved non-academics felt they should be in the research process. Some wanted to engage regularly and contribute to funding bids, viewing research as an integral part of their role, whilst others felt they lacked capacity for research involvement. No clear methods for building collaborative relationships were identified; however, willingness to engage (in non-academics) and reputation (of academics) were mentioned by study participants.

### Challenges of engagement in the research process

Challenges relating to building and maintaining relationships, data/information, getting evidence into action, culture and systems/structures were identified that made collaborative working and engaging in the research process difficult (Supplementary file 2).

#### Building and maintaining relationships

Establishing and building collaborative relationships was identified as time consuming and challenging for several participants, particularly those who lacked skills or confidence in networking. This included identifying and getting partners on board, connecting with people with shared interests and building multidisciplinary teams.

Maintaining relationships was also identified as a challenge. There was a recognition that partnerships do not always work for a range of reasons, for example, having incompatible disciplinary views. Academics found it difficult to manage relationships when the results were not what stakeholders expected. Conflicting priorities between practitioners and academics were also evident, for example, a practitioner may wish to deliver and evaluate multiple interventions in one setting, whereas an academic may prefer to deliver and evaluate only one. When research interests change and collaborators move roles, this also poses a challenge for maintaining working relationships. Finally, some interviewees noted that participants were sometimes involved in research without benefiting from their involvement or without an understanding of their needs. Identifying ways of engaging the public in research in mutually beneficial ways was identified as a priority moving forward.

“*… often the people actually doing the work on the ground, have an initial, what’s going on? You’re getting in the way of my work because of course you have to get the funding, you have to get the baseline data in if it’s an intervention and often that means you have to say ‘yes, we want you to do the work but not yet’, which can be maddening if you’ve got a community that wants something to happen, you’ve engaged with them, you’ve got the funding to actually do the intervention, you want to get on with it, not hold off whilst some academics fuss about getting the data before you do anything.*” (Academic, participant 2)

#### Data/information

Participants described a lack of data sharing, lack of usable data, small sample sizes and poor-quality research as issues. In addition, duplication of research efforts due to a lack of communication and siloed working were observed. Ongoing research gaps were identified by non-academics that were not being addressed by academics due to a perceived lack of interest. Barriers to engaging in research, including a lack of knowledge of the research system, were identified by some non-academics. Similarly, others expressed difficulties in navigating internal policies with other organisations to collaborate on projects.

“*People start scuttling back into silos… there’s a lot of people doing stuff but it’s just not being managed very well and the end result of that is that the community, I don’t know, it doesn’t serve anybody well, so if research can actually help to show efficacy of what really works and then people can pull together on a wider scale round that and say let’s work together.*” (Practitioner, participant 4)

#### Evidence into action

Translating research into policy and practice was consistently identified by the participants as a challenge. Academics found it challenging to demonstrate impact. Similarly, translating research into practice was identified by non-academics as a challenge, particularly where funding for research translation or implementation was required. Some non-academics identified a disconnect between research and application and felt that research is not giving new insights into practice.

“*Often what you'll find is either a researcher or an academic will produce the classic piece of research and then think why is this not being acted on? Why has this not got its way into policy or practice? And why? It's because policy-makers or practitioners don't know about it, or because the timing's wrong. Or the flipside of it is the window of opportunity for a policy-maker will open and they'll be looking to evidence and knowledge base. If they haven't got that relationship and they don't know what evidence or research is out there or don't value it then they won't go looking for it. They won't be able to find it.*” (Practitioner, participant 5)

#### Culture

There was a perception amongst the participants that non-academic research is less rigorous than academic research, that some non-academics lack objectivity, and that academics are sceptical of non-academic research. Academic research was generally perceived to be of good quality, better resourced and of a high reputation.

There were uncertainties in relation to how much involvement non-academics should have in the research process and the role of non-academics in shaping the research agenda. Lack of time and funding were key barriers for non-academic involvement in research. Understanding and applying research findings to practice were further challenges for non-academics, with some feeling that they lacked research skills, which was a barrier to contributing to and using research.

The perception of the quality of research evidence was an identified challenge in relation to use of research evidence, with academics noting an ongoing perception in public health that randomised controlled trials are considered gold standard evidence.

“*… there needs to be a recognition in public health that natural experiments are sometimes the only possible and indeed the only appropriate ecologically valid way to understand relationships between use of the outdoor environment and health. Therefore, the weaknesses that inevitably in experimental design or associated with those, should not be seen as the research itself being of poor quality.*” (Academic, participant 2)

#### System/structure

Participants felt that funding processes can be overly selective and that changes in processes were difficult to keep up with. Time spent securing funding was an identified challenge and it was noted that academic funding timelines do not work with non-academic timelines. Additional funding challenges included funding not always being placed where it is most needed, funding favouritism (organisations preferring to fund specific researchers they know), government funding for research being used to justify pre-planned work, a lack of resources to commission or to fund work that needs to be done, lack of resources to fund the scale of research needed, or unwillingness to fund projects with long-term follow-up. Some participants also felt that academics were more inclined to submit ‘safer’ projects, which were less reflective of stakeholder need, due to the funding process and job security. Academic involvement in projects was felt to be expensive, particularly for government or not-for-profit-funded projects. Some non-academics wanted to influence research agendas and the nature of research call documents but were unsure about how they could do this. Finally, there was a recurring concern amongst non-academics that time for their involvement in research projects is often not funded, yet non-academics are expected to make large time commitments to communicate and collaborate with researchers to achieve project goals. In contrast, one academic felt that non-academics were happy to be involved in research without their time being funded. “[We] *never saw a penny of the research funding … it was really difficult for us in terms of just creating the time and the space to engage. But we did see a value in that*” (Practitioner, participant 1).

Non-academics noted that the academic research process was slow and felt frustrated about having to wait for publication to see results and the time it takes to obtain research approvals. Competing tasks or projects were felt to compound this. This presented a challenge for applying research findings or committing to engaging in research when stakeholder priorities are likely to change before funded research projects could begin.

Getting evidence into action was identified as a challenge and, equally, it was felt that research is not always informed by policy and practice or useful for this audience. A rapidly changing political context, in contrast to academic timescales, was also found to present a challenge for the generation of impactful and usable research. A need for skills to bridge the gap between academics and non-academics was identified to ensure that research is informed by policy and practice and vice versa. Finally, one participant expressed concerns that the government suppressed unfavourable research findings, where the findings were deemed to conflict with current political interest.

### Suggestions for change

Suggestions were put forward to address the challenges identified. These included changes to the funding system, methodologies, evidence into policy and practice, and to methods for collaborative working.

#### The funding system

A proposal was made that funders should actively and regularly consider ways to improve the funding system, for example, by funding non-academic involvement in research and involving non-academics in shaping the research that is commissioned.

“*… it feels tremendously farfetched, but if ever there was a mechanism whereby* [non-academic] *organisations … could be supported centrally through the research councils or research funders, to almost give us a mandate to go and work with the research community on projects. So almost a … I don't know what you'd call it really, but an allocation that enabled us to engage with bits of the research community would be an amazing solution to it, I think. I've never had that conversation with the research councils. But if they said right, there's enough funding to have a dedicated person who just works with the research community, then we could immediately go and find 20 projects that would really welcome our involvement at no cost to them. That we could really support and work with and hopefully improve the research outcomes from those projects.*” (Practitioner, participant 1)

#### Methodologies

Suggestions for changing research methodologies to support collaborative working and use of research included measuring relevant outcomes at appropriate time points, preparing contingency plans and using research approaches that engage meaningfully with participants. Citizen science and community action research were proposed as potential methods of facilitating empowering community engagement.

“*Working with community groups, with research mentors, to help them identify their own research questions and then to develop and design research programmes to answer the questions themselves … that has a huge power because it puts the hands of the research question and then taking the research in the community, so it’s something that they are doing themselves and doing with research partners, rather than being the subject of research and research being done to them.*” (Practitioner, participant 9)

#### Evidence into policy and practice

Suggestions to enhance the use of evidence in policy and practice included obtaining a clearer picture of what government needs to inform policy, training postgraduate students on creating impactful research, effective dissemination of research that is engaging and understandable to non-academics, developing skills in translation of research findings, and roles that bridge the gap between academic and non-academics such as knowledge-exchange brokers.

#### Better collaboration between academics and non-academics

Several participants wanted to see more meaningful collaboration between academics and non-academics. Participants felt that this could enable non-academics to shape research priorities and designs, enable data sharing and find out about new research. However, as previously noted, some participants were satisfied with their level of involvement or felt that involvement in research was not part of their job. Skill development in forming collaborative relationships and networking was identified as a need amongst some participants.

## Discussion

NCDs are a growing public health concern and, given the estimated high levels of research wastage and lack of viable solutions to tackle NCDs, there is a need for honest reflections on the public health research process. There is also a need to recognise the research system as a complex system, which interacts with and is influenced by multiple other systems, for example, funding, local and national government, and education systems. Framing and viewing the research system using systems thinking can help to identify and develop sustainable approaches to improving the research process [30]. This study qualitatively explored stakeholders’ experiences of engaging in the research process in outdoor space and NCD prevention. Enablers, challenges and suggestions for improvement to the research process were identified. The findings build on previous research on partnerships [14] and provide a starting point to enhance the research–policy–practice relationship using systems thinking, ultimately to tackle the growing burden of NCDs.

Carefully planned and resourced research, including team members with appropriate skills, was found to facilitate stakeholder engagement in the research process. Consistent with previous research, carefully planned and managed research with attention to context and culture, and where the research team was effective, respective and communicative, was found to enable the use of research evidence and facilitate positive experiences of engagement in the research process [31, 32]. Embedding collaborative research into routine practice could be enabled through policy changes at the research and funding level. For example, all research (where appropriate) at the Scottish Collaboration for Public Health Research and Policy, University of Edinburgh, involves collaborations with policy-makers, practitioners or community members with the collective goal of improving public health. This contributes to building mutually beneficial relationships, where collaborators and researchers can propose research questions, conduct research and implement findings. Considering the feasibility of introducing policies aimed at enabling collaborative research practices, whilst maintaining researcher objectivity and an ability to produce revolutionary scientific advances, has the potential to address the challenges identified in this study across the identified sub-themes. Such strategies could develop the learning of both stakeholders and researchers, assist with the translation of research findings, and lead to the production of research that better meets the needs of community members [12, 31].

Policies and strategies aimed at increasing collaborative research are unlikely alone to transform stakeholder engagement in the research process and it is important that any policies in relation to collaborative working sit within a broader suite of strategies to address the challenges identified. Challenges ranged from factors that could be relatively straightforward to address (e.g. collaborators moving roles) and public health capacity-building, to complex, systemic factors required to better align research, policy and practice systems, which adds to previous research findings [33, 34]. Systemic changes would arguably be more difficult to address (e.g. the nature of research funding and the academic system). It is important that any solutions address identified barriers for non-academic participation, such as costing the time of non-academics in research funding bids for collaborative projects where appropriate. Some potential solutions to some of these challenges, based on our own experiences of the research system, are outlined in Table 1. Implementing and evaluating these potential solutions and identifying and piloting additional strategies to address the identified challenges using systems thinking could provide further insight to improve the research–policy–practice relationship and make the research system more accessible to non-academics.

**Table 1:** Potential solutions to enhance stakeholder engagement in the research process

Challenges	Potential solutions^a^
Building and maintaining relationships	● Dedicated communication channels and strategies amongst academic and non- academic stakeholders to share ideas, priorities and challenges such as regular newsletters, forums, social media or networks that encourage two-way exchange of ideas and insights
	● Collaborative priority-setting approaches within government, non-government and community-based health sectors, facilitated by multi-stakeholder coalitions, or collaborative meetings to enhance collaborative decision-making across research, policy and practice; for example, in Scotland, expert working groups have been established to advise government on key topics such as the Women and Girls in Sport Advisory Group (https://www.gov.scot/groups/women-and-girls-sport-advisory-board/)
	● Dedicated knowledge exchange services/programmes with specialist skills/training within or external to universities to connect researchers with policy or practice organisations and enable research to be more routinely informed by policy and practice and vice versa [35–37]
	● Create online repositories for lay audiences to summarise research to maximise dissemination and impact; for example, the SHaRE project at the University of Edinburgh is an online repository where practitioners and policy-makers can access walking for health research conducted in/relevant to Scotland (http://www.sparc.education.ed.ac.uk/share/)
Data/information	● Make data publicly available in relevant repositories where appropriate
	● Publish accessible summaries of research findings suitable for the target audience, such as policy briefs, lay summaries or infographics, and identify ways of successfully disseminating these summaries to the target audience (e.g. through dedicated communication channels mentioned previously)
Evidence into action	● Facilitation of consultation and action through professional associations related to public health; for example, Scotland’s Public Health Evidence Network was set up by NHS Health Scotland alongside key academic and non-academic partners, including the Scottish Collaboration for Public Health Research and Policy and Healthcare Improvement Scotland. The work of the Public Health Evidence Network is directly guided through consultation with policy-makers, who play a central role throughout
	● Professional associations for advocacy of the need for evidence into practice, using coalitions mentioned above to help with the translation of evidence into action
Culture	● Celebrate and incentivise initiatives, projects and outputs including multi-stakeholder research teams, for example, through academic promotion processes
System/structure	● Policy and systems change in both funding and research sectors to require or encourage academic and non-academic collaboration such as funders requiring evidence of stakeholder engagement in developing research ideas in grant applications or funding systems informed by evidence that address areas of greatest health and social need
	● Encourage and incentivise non-academics to publish research findings to build rigour of both evidence-based practice in public health and to help to collaboratively identify, on the ground, needs and opportunities from public health organisations and practitioners from relevant journals for non-academic publishing
	● Engagement of practitioners and other non-academic stakeholders through relevant adjunct or similar appointments in university departments (and vice versa) to facilitate research–policy–practice engagement

^a^Based on study results, co-authors experiences and research (where available)

Whilst in this paper we have discussed non-academic partners as practitioners, policy-makers and other professionals involved in public health, naturally, the community is also an integral stakeholder; this has been well noted in previous reports [19, 38] and is further supported by these findings. Our research suggests a need for a wider discussion on how to engage the public in research in ways that are meaningful and empowering, enabling members of the public to be involved in the strategic direction of research as well as the conduct, analysis and implementation of findings. Citizen-led approaches, such as citizen science, may enable the public to develop skills and use these skills and the data generated to improve their local communities. The development of these and other citizen-led research approaches has been suggested to have the potential to transform public health science and policy-making [8] and should be an ongoing discussion for the research, policy and practice community.

### Strengths and limitations

A broad range of perspectives and experiences, including representatives from policy, practice, research, knowledge brokers and funders, were included to outline the challenges and enablers to stakeholder engagement in the research process. This contributed to rich data addressing the research questions. Whilst a broad range of perspectives were included, findings are limited to a relatively small sample of professionals (*n* = 20) from a broad range of organisations with interests in outdoor space and NCD prevention in a United Kingdom context. Thus, the findings may not be transferable to other countries or disciplines where differences in collaborative relationships or the research process may exist. Including perspectives from members of the public and academic journal editorial staff could have provided additional insights.

## Conclusions

This study identified a range of facilitators and challenges in relation to stakeholder engagement in research across multiple levels. Challenges related to forming and maintaining collaborative relationships, accessibility of data/information, getting evidence into action, culture, and systemic and structural challenges. The findings of this study add to existing literature on the importance of collaborative research for research impact; however, the identified barriers highlight structural, cultural and individual barriers that need to be addressed to enable effective collaborative relationships between research, policy and practice. Developing and piloting approaches in collaboration with stakeholders to address the identified challenges of stakeholder engagement using systems thinking could contribute to positively changing the research system and how research, policy and practice intertwines, and ultimately improve research outcomes and reduce NCDs.

## Supplementary information

**Additional file 1.** Fishbone diagram.

**Additional file 2: Supplementary file A.** Basic coding framework.

## Data Availability

The datasets used and/or analysed during the current study are available from the corresponding author on reasonable request.

## Abbreviation

NCDs: non-communicable diseases
