# Supplementary material for: Stakeholders’ experiences of the public health research process: time to change the system?
Source: Health Res Policy Syst. 2020 Jul 18;18:83. doi: 10.1186/s12961-020-00599-5 (PMC7368787; doi:10.1186/s12961-020-00599-5)
Supplement: Supplementary file 1 — Additional file 1. Fishbone diagram. [file 12961_2020_599_MOESM1_ESM.pdf]

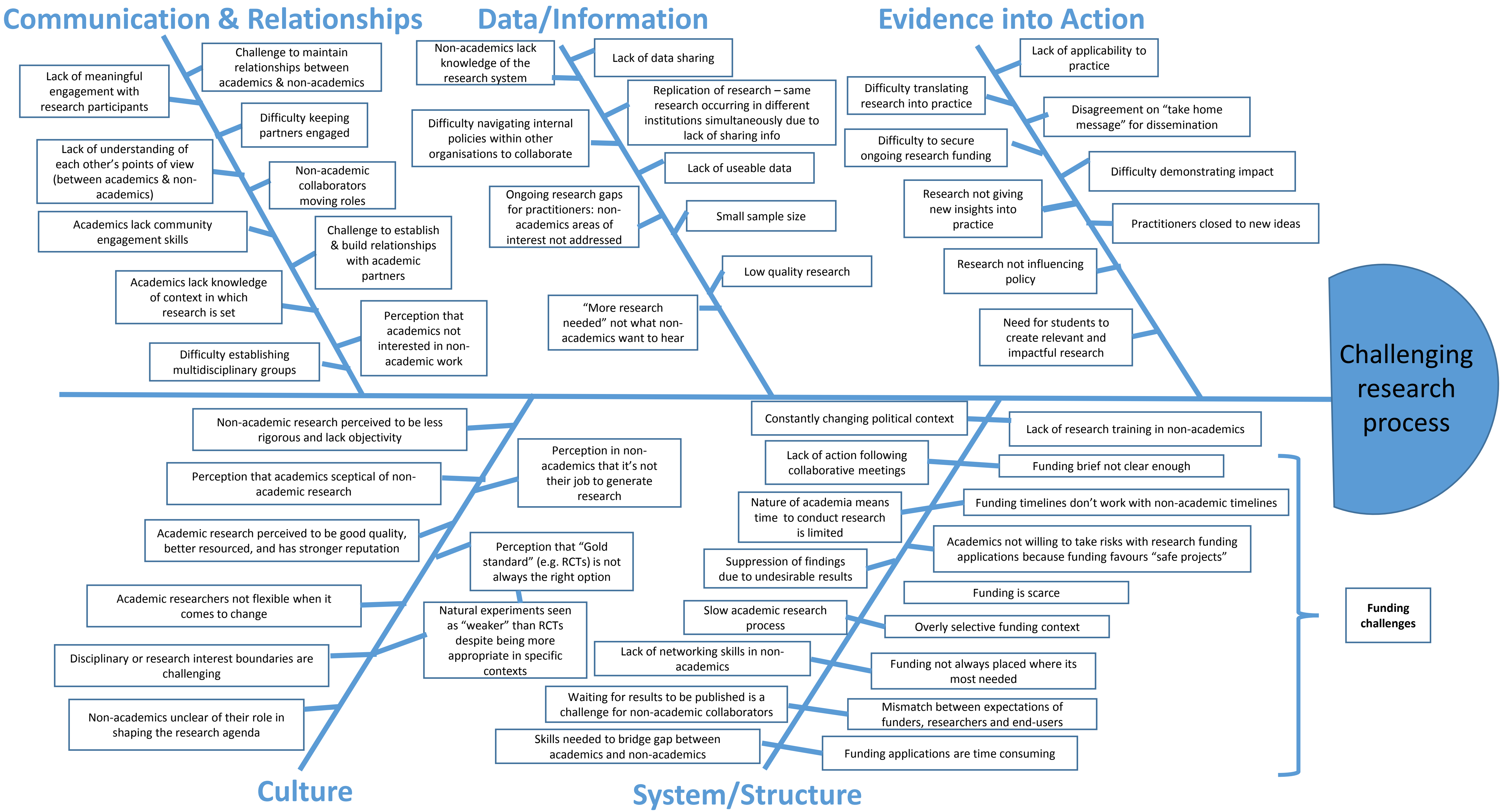
Communication & Relationships
Challenge to maintain relationships between academics & non-academics
Lack of meaningful engagement with research participants
Difficulty keeping partners engaged
Lack of understanding of each other's points of view (between academics & non-academics)
Non-academic collaborators moving roles
Academics lack community engagement skills
Challenge to establish & build relationships with academic partners
Academics lack knowledge of context in which research is set
Perception that academics not interested in non-academic work
Difficulty establishing multidisciplinary groups
Data/Information
Non-academics lack knowledge of the research system
Lack of data sharing
Replication of research – same research occurring in different institutions simultaneously due to lack of sharing info
Difficulty navigating internal policies within other organisations to collaborate
Lack of useable data
Ongoing research gaps for practitioners: non-academics areas of interest not addressed
Small sample size
Low quality research
"More research needed" not what non-academics want to hear
Evidence into Action
Lack of applicability to practice
Difficulty translating research into practice
Disagreement on "take home message" for dissemination
Difficulty to secure ongoing research funding
Difficulty demonstrating impact
Research not giving new insights into practice
Practitioners closed to new ideas
Research not influencing policy
Need for students to create relevant and impactful research
Challenging research process
Non-academic research perceived to be less rigorous and lack objectivity
Perception in non-academics that it's not their job to generate research
Perception that academics sceptical of non-academic research
Academic research perceived to be good quality, better resourced, and has stronger reputation
Perception that "Gold standard" (e.g. RCTs) is not always the right option
Academic researchers not flexible when it comes to change
Natural experiments seen as "weaker" than RCTs despite being more appropriate in specific contexts
Disciplinary or research interest boundaries are challenging
Non-academics unclear of their role in shaping the research agenda
Culture
Constantly changing political context
Lack of research training in non-academics
Lack of action following collaborative meetings
Funding brief not clear enough
Nature of academia means time to conduct research is limited
Funding timelines don't work with non-academic timelines
Academics not willing to take risks with research funding applications because funding favours "safe projects"
Suppression of findings due to undesirable results
Funding is scarce
Slow academic research process
Overly selective funding context
Lack of networking skills in non-academics
Funding not always placed where its most needed
Waiting for results to be published is a challenge for non-academic collaborators
Mismatch between expectations of funders, researchers and end-users
Skills needed to bridge gap between academics and non-academics
Funding applications are time consuming
Funding challenges
System/Structure
