# Supplementary material for: Stakeholders’ experiences of the public health research process: time to change the system?
Source: Health Res Policy Syst. 2020 Jul 18;18:83. doi: 10.1186/s12961-020-00599-5 (PMC7368787; doi:10.1186/s12961-020-00599-5)
Supplement: Supplementary file 2 — Additional file 2: Supplementary file A. Basic coding framework. [file 12961_2020_599_MOESM2_ESM.docx]

| **Code level 1** | **Code level 2** | **Sources** | **Refs** |
| --- | --- | --- | --- |
| **Impact into action (challenges)** |  |  |  |
|  | **Demonstrating impact** | 2 | 2 |
|  | **Low research quality** | 1 | 3 |
|  | **Ongoing research gaps** | 2 | 3 |
|  | **Research consensus on dissemination** | 1 | 1 |
|  | **Replication of research** | 2 | 3 |
|  | **Research not newsworthy** | 1 | 1 |
|  | **Suppression of findings** | 1 | 2 |
|  | **Translating research into practice**   - **Disconnect between research and application** - **No new insights** - **Securing funding** - **Practitioners not open to new ideas** | 9 | 14 |
| **Nature of academia (challenges)** |  |  |  |
|  | **Slow research process** | 5 | 5 |
|  | **Researchers opt for safer projects for progression** | 2 | 3 |
|  | **Lack of time** | 4 | 4 |
|  | **Perceptions of gold-standard evidence** | 4 | 5 |
| **Non-academic involvement** |  |  |  |
|  | **How non-academics are involved** | 10 | 12 |
|  | **Benefits of non-academic involvement to research** | 16 | 29 |
|  | **Benefits of non-academic involvement to practice** | 9 | 11 |
|  | **How non-academics want to be involved** | 13 | 35 |
| **Challenges of engagement** |  |  |  |
|  | **Challenges of non-academic involvement** | 12 | 25 |
|  | **Barriers for non-academic involvement** | 8 | 15 |
| **Perceptions of academic vs non-academic research** |  |  |  |
|  | **Perceptions of academic research** | 8 | 16 |
|  | **Perceptions of non-academic research** | 8 | 7 |
| **Funding environment (challenges)** |  |  |  |
|  | **Scarce resources** | 8 | 13 |
|  | **Funding process** | 6 | 6 |
|  | **Practitioners time not funded** | 5 | 8 |
|  | **Practitioner time spent on project extensive** | 1 | 2 |
|  | **Ensuring calls have practice element** | 1 | 1 |
|  | **Clarity of brief** | 1 | 1 |
| **Perspectives of good research** |  |  |  |
|  | **Generates new insights** | 8 | 11 |
|  | **Impactful** | 16 | 40 |
|  | **Methodologically sound** | 4 | 9 |
|  | **Outcomes**   - **Communicate impact** - **Further funding** - **Results inform decision-making** - **Generate understanding** - **Inform policy** | 16 | 47 |
| **Suggestions for change** |  |  |  |
|  | **Funding system**   - **Funding non-academics time** - **Streamlined processes** | 6 | 9 |
|  | **Improve research impact and translation**   - **Better use of evidence to shape policy** - **Clearer picture of what government needs to inform policy** - **Effective research dissemination** - **Knowledge translation roles** - **Enhance skills in research translation** - **Train students to create usable research** | 6 | 11 |
|  | **Improved research design**   - **Engage non-academics in meaningful ways** - **Risk management/contingency plans** | 9 | 19 |
